# Seroprevalence and associated risk factors of *Toxoplasma gondii* infection in yaks (*Bos grunniens*) on the Qinghai–Tibetan Plateau of China

**DOI:** 10.1051/parasite/2021043

**Published:** 2021-05-18

**Authors:** Tao Sun, Sajid Ur Rahman, Jinzhong Cai, Jiangyong Zeng, Rongsheng Mi, Yehua Zhang, Haiyan Gong, Hongcai Ma, Yan Huang, Xiangan Han, Quan Zhao, Zhaoguo Chen

**Affiliations:** 1 Key Laboratory of Animal Parasitology of Ministry of Agriculture, Laboratory of Quality and Safety Risk Assessment for Animal Products on Biohazards (Shanghai) of Ministry of Agriculture, Shanghai Veterinary Research Institute, Chinese Academy of Agricultural Sciences Shanghai 200241 PR China; 2 Animal Science and Technology College, Jilin Agricultural University Changchun 130118 Jilin PR China; 3 Veterinary Research Institute, Qinghai Academy of Animal Sciences and Veterinary Medicine, Qinghai University, State Key Laboratory of Plateau Ecology and Agriculture Xining 810016 Qinghai PR China; 4 Tibet Livestock Research Institute, Tibet Academy of Agriculture and Animal Science Lhasa 850009 Tibet PR China

**Keywords:** *Toxoplasma gondii*, Yak, Seroprevalence, Risk factors, Qinghai–Tibetan Plateau

## Abstract

*Toxoplasma gondii* is an intracellular parasite that is extensively prevalent globally. Studies have indicated the presence of *T. gondii* infection in animals in some provinces of China, but little is known about *T. gondii* infection in yaks (*Bos grunniens*) on the Qinghai–Tibetan Plateau. In the current study, to determine the seroprevalence and associated risk factors of *T. gondii*, a total of 2784 serum samples were collected from 18 different sampling sites in eight counties of the Qinghai and Tibet regions of China from 2018 to 2019. Serum antibodies against *T. gondii* were detected in 261 yaks (9.38%) via enzyme-linked immunosorbent assay (ELISA). We found that seroprevalence differed significantly among different counties (ranging from 5.41% in Gangcha to 19.79% in Datong), by year in the Tibet Autonomous Region (from 2.34% in 2018 to 13.24% in 2019), and by age (from 5.59% in 0 < year ≤ 1 to 11.76% in year > 7) (*p* < 0.05). Climate, geographical conditions, and age are the main factors influencing *T. gondii* infection in yaks in these regions. Therefore, our study provides a data reference for public health and prevention of yak toxoplasmosis.

## Abstract (fr)

*Toxoplasma gondii* est un parasite intracellulaire largement répandu dans le monde. Des études ont indiqué la présence d’une infection par *T. gondii* chez les animaux dans certaines provinces de Chine, mais on connaît peu l’infection par *T. gondii* chez les yaks (*Bos grunniens*) sur le plateau Qinghai–Tibet. Dans la présente étude, pour déterminer la séroprévalence et les facteurs de risque associés de *T. gondii*, un total de 2784 échantillons de sérum ont été prélevés sur 18 sites d’échantillonnage différents dans huit comtés des régions du Qinghai et du Tibet en Chine entre 2018 et 2019. Des anticorps sériques contre *T. gondii* ont été détectés par dosage immuno-enzymatique (ELISA) chez 261 yaks (9,38 %). Nous avons constaté que la séroprévalence différait considérablement entre les différents comtés (allant de 5,41 % à Gangcha à 19,79 % à Datong), d’une année à l’autre dans la région autonome du Tibet (de 2,34 % en 2018 à 13,24 % en 2019), et par âge (de 5,59 % pour les animaux de moins d’un an à 11,76 % pour ceux âgés de plus de 7 ans) (*p* < 0,05). Le climat, les conditions géographiques et l’âge sont les principaux facteurs influençant l’infection à *T. gondii* chez les yaks de ces régions. Par conséquent, notre étude fournit des données de référence pour la santé publique et la prévention de la toxoplasmose du yak.

## Introduction

*Toxoplasma gondii* is an obligate intracellular parasite that infects various host species worldwide, including yak (*Bos grunniens*) [6]. This pathogen can be assimilated by oocyst-contaminated vegetables, drinking water, fruits, or by the consumption of undercooked meat contaminated with dormant cysts, as well as by trans-placental tachyzoite transmission [2, 21, 22]. In humans, *T. gondii* infection usually does not show any obvious symptoms; however, in patients with weak immune function or immunodeficiency, such as acquired immunodeficiency syndrome (AIDS) patients, infection can lead to serious diseases including epilepsy, encephalitis, retinitis, and even death with virulent strains of *T. gondii* [14, 25, 26]. Acute infection is followed by asymptomatic dormant infection when parasites develop in numerous organs, such as the skeletal/cardiac muscles, retina, and brain parenchyma [27]. Dormant infection can reboot in immunocompromised patients, with alteration of dormant bradyzoites into duplicating tachyzoites leading to substantial morbidity and 100% mortality [20]. Previous studies have shown that *T. gondii* infection during pregnancy can lead to miscarriage, premature birth, birth defects, or intellectual disability [7].

The domestic yak (*Bos grunniens*) is a long-haired domesticated bovid found throughout the Himalayan region of the Tibetan Plateau, Northern Myanmar, Sichuan, Yunnan, and as far north as Mongolia and Siberia. Yaks live in the cold climates and high plateaus (over 3000 m) of China, Bhutan, Nepal, Mongolia, Russia, India and other countries [10, 16]. It has been estimated that about 90% of the world’s yaks live on the Qinghai–Tibetan Plateau in China [16]. Milk, meat, faeces and wool of yaks are closely related to the lives of local residents [10]. Yaks graze freely on the Qinghai–Tibetan Plateau, living with other wild animals and domestic livestock. They also share pasture grass and water with a large number of Plateau pika (*Ochotona curzoniae*) and Qinghai vole (*Microtus fuscus*) in the region and these species have been confirmed to carry *T. gondii* [28]. Rodents and small animals are also intermediate hosts of *T. gondii,* and carnivores such as the Snow leopard (*Panthera uncia)*, Pallas’s cat (*Otocolobus manul*), Siberian weasels (*Mustela sibirica*), cats, and dogs prey on them. This leads to multiple settings in which yaks can come into close contact with *T. gondii* oocysts.

Nowadays, serological assays are widely used for the clinical diagnosis of toxoplasmosis and these include the indirect hemagglutination test (IHA), indirect fluorescent antibody test (IFAT), and enzyme-linked immunosorbent assay (ELISA) [4, 23, 28]. Previously, studies have reported that the seroprevalence of *T. gondii* infection in yaks in the Qinghai–Tibetan Plateau of China is between 2.3% and 35.1% [15, 17, 18]. However, the prevalence rate differed depending on geographical location. In remote areas of the plateau, *T. gondii* infection of yaks can cause greater economic losses and threaten other animals and human health.

In order to enrich the information on the prevalence and distribution of *T. gondii* infection in yaks in the Qinghai–Tibet Plateau, sera of yaks were collected between 2018 and 2019 and *T. gondii* antibodies were monitored. Our results demonstrate the dynamic epidemic situation of toxoplasmosis in yaks in the Qinghai–Tibet Plateau, and provide support for control of *T. gondii* infection in yaks and food safety.

## Materials and methods

### Ethics statement

The experimental protocol was approved by the ethics committee of Shanghai Veterinary Research Institute, Chinese Academy of Agricultural Sciences, approval number (SHVRI-SZ-201811015-03).

### Sampling sites and blood collection

**Figure 1:**
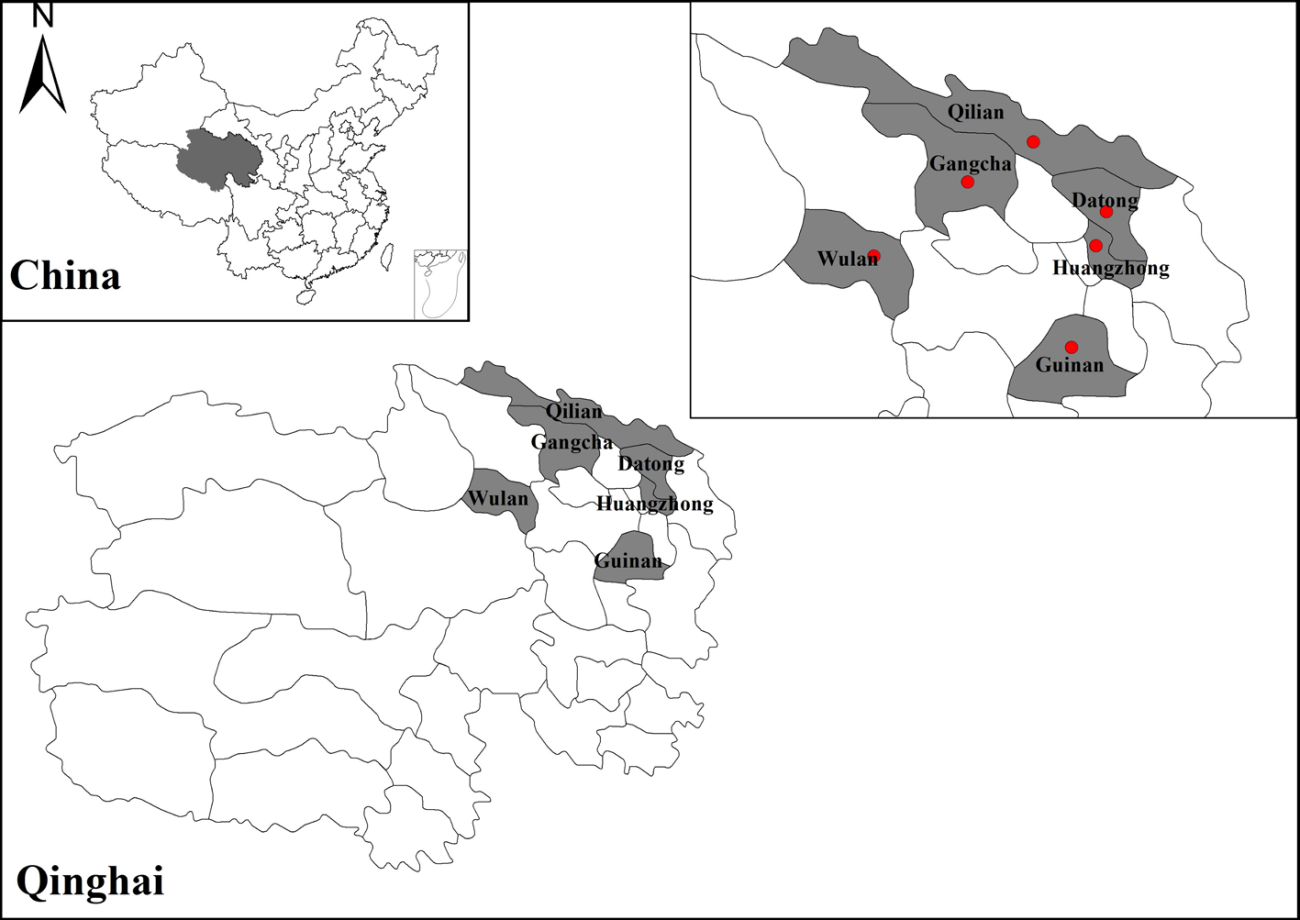
Map of sampling counties in Qinghai in this study. The red dots show the sampling sites.

**Figure 2:**
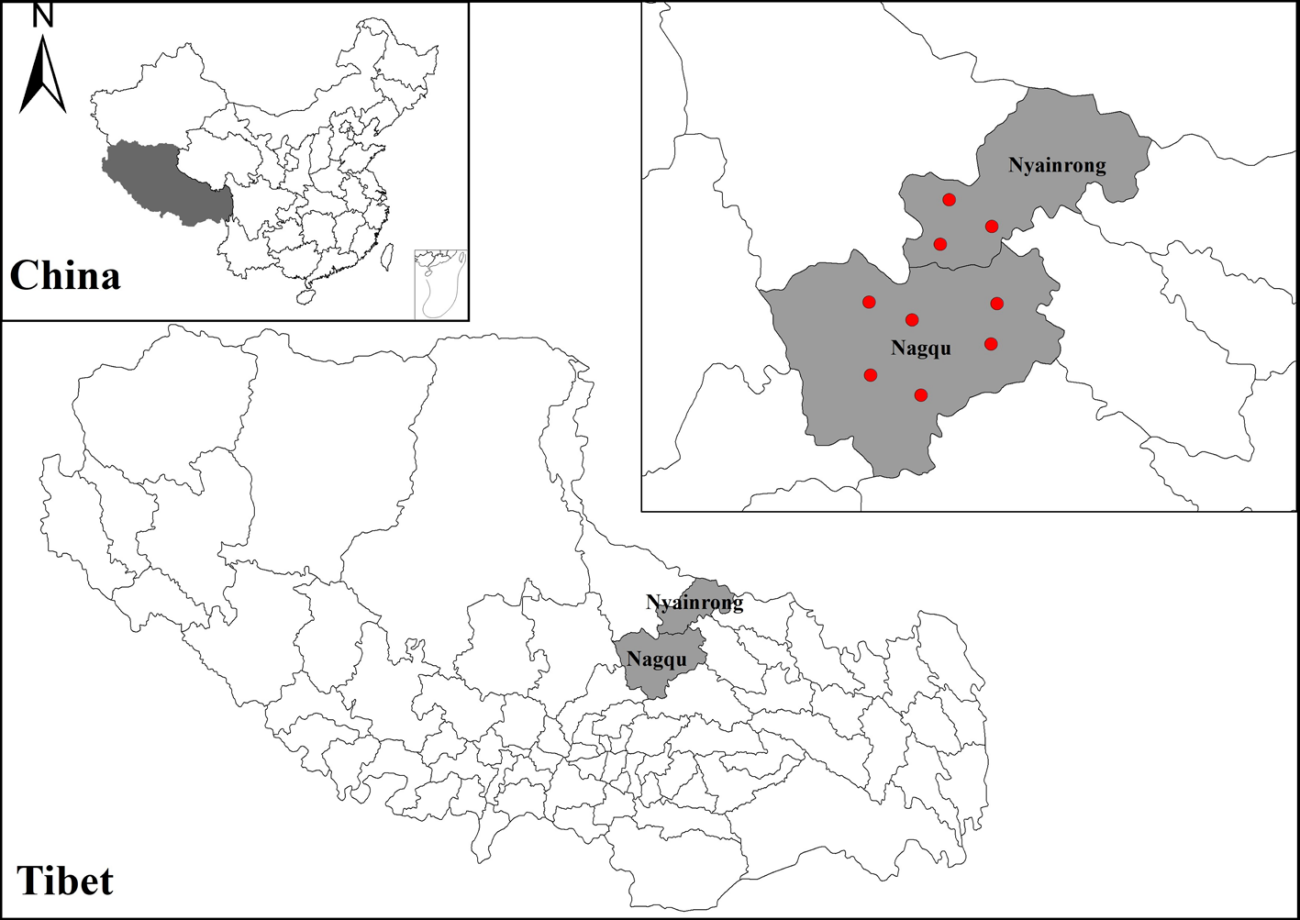
Map of sampling counties in Tibet in this study. The red dots show the sampling sites.

The blood samples of yaks used in this study were collected from six counties (Wulan, Gangcha, Huangzhong, Guinan, Qilian, and Datong) in Qinghai Province with a latitude of 35°09′–38°35′N, and longitude of 97°01′–101°56′E (Fig. 1), and two counties (Seni and Nyainrong) in the Tibet Autonomous Region with latitude of 30°31′–33°24′N, and longitude of 91°12′–93°56′E (Fig. 2). A total of 2784 blood samples were collected from the jugular vein of yaks from 2018 to 2019. Disposable blood collection needles (LuTai, ST1021, Shandong, PR China) and vacuum blood collection tubes (BD-Pharmingen, 367820, USA) were used to collect blood samples. Yak owners were asked to provide information on the age and sex of the animal using a questionnaire. After collection, blood samples were centrifuged at 1 500× *g* for 15 min and sera were separated and transferred to 1.5 mL Eppendorf tubes. The serum samples were stored at −80 °C until use.

### Detection of *T. gondii* antibodies

Antibodies for *T. gondii* were determined using a commercial *T. gondii* IgG enzyme-linked immunosorbent assay (ELISA) Kit (Indical Bioscience, Leipzig, Germany). Both positive and negative control sera were available in the kit. The diluted serum sample (1:100) was incubated for 60 min at room temperature in the assay well and then washed three times. Then 100 μL of conjugate was added to each well and incubated for 30 min at room temperature. The plate was again washed three times and the chromogenic enzyme substrate was added at a concentration of 100 μL and incubated for 10 min at room temperature. A stop solution of 100 μL was then added to each well to stop the reaction. Finally, the optical density values (OD) of each well were measured using an ELISA plate reader (Epoch Bio-Tek, Winooski, VT, USA) at 450 nm.

The ratio (*S*/*P*) values were calculated according to the following equation:

(1) $$\frac{S}{P}=\frac{{\mathrm{OD}}_{450}\left(\mathrm{sample}\right)-\mathrm{mean}{\mathrm{OD}}_{450}\left(\mathrm{Negative}\mathrm{Control}\right)\mathrm{value}}{\mathrm{mean}{\mathrm{OD}}_{450}\left(\mathrm{Positive}\mathrm{Control}\right)\mathrm{value}-\mathrm{mean}{\mathrm{OD}}_{450}\left(\mathrm{Negative}\mathrm{Control}\right)\mathrm{value}}.$$

The serum was considered positive for *T. gondii* if *S*/*P* ≥ 0.2.

### Statistical analysis

Statistical analysis was performed using chi-square test with IBM SPSS, v19.0 (SPSS Inc., Chicago, IL, USA). Differences were considered statistically significant when *p* < 0.05.

## Results

### Seroprevalence of *T. gondii* in yaks of different locations

Serum samples from 2784 yaks were examined via ELISA, and 261 tested positive for *T. gondii* antibodies, representing a seroprevalence rate of 9.38%. Among them, the prevalence of *T. gondii* in yaks in Qinghai Province was 8.63% (95/1101; 95% CI, 7.0–10.5), but, in the Tibet Autonomous Region the prevalence rate was 9.86% (165/1683; 95% CI, 8.4–11.3). No significant difference was found between province and region (*p* > 0.05).

As shown in Table 1, seroprevalence in different counties ranged from 5.41% to 19.79%, showing a statistically significant difference (*p* < 0.05). Across 18 sites, ELISA results demonstrated that the highest positive rate occurred in Datong Ranch (19.79%, 19/96; 95% CI, 12.4–29.2); however, the lowest prevalence rate was found in Qilian Ranch (4.19%, 7/167; 95% CI, 1.7–8.5), showing a significant difference (*p* < 0.05) between these sites (Table 1).

**Table 1:** Prevalence of *T. gondii* in yaks in different provinces, counties and sampling sites on the Qinghai-Tibetan Plateau.

Provinces	Counties	Positive rates% (No. positive/No. samples)	95% CI	Sampling sites	Positive rates% (No. positive/No. samples)	95% CI
Qinghai	Datong	19.79(19/96)^a^	12.4–29.2	Datong Ranch	19.79(19/96)^a^	12.4–29.2
	Gangcha	5.41(4/74)^bc^	1.5–13.3	Qingqingcao Ranch	5.41(4/74)^de^	1.5–13.3
	Guinan	7.06(19/269)^bc^	4.3–10.8	Laozhaxi Ranch	7.06(19/269)^de^	4.3–10.8
	Huangzhong	14.77(13/88)^ab^	9.1–23.9	Slaughter houses	17.02(8/47)^abc^	7.7–30.8
				Fengtai Ranch	12.20(5/41)^abcd^	4.3–12.1
	Qilian	6.01(22/366)^c^	3.8–9.0	Qilain Ranch	4.19(7/167)^e^	1.7–8.5
				Warinai Ranch	7.54(15/199)^de^	9.3–13.4
	Wulan	8.65(18/208)^bc^	5.2–13.3	Chaidamu Ranch	0.00(0/11)^abcde^	0.0–28.5
				Tongpu Ranch	9.14(18/197)^bcde^	5.0–13.5
	Subtotal	8.63(95/1101)	7.0–10.5		8.63(95/1101)	7.0–10.5
Tibet	Seni	9.98(80/802)^b^	8.0–12.3	Dasa Village	8.07(13/161)^cde^	4.4–13.4
				Gaerde Ranch	9.65(11/114)^bcde^	4.9–16.6
				Maqing Village	12.34(19/154)^abcd^	7.6–18.6
				Mufa Ranch	5.00(5/100)^de^	1.6–11.3
				Namaqiegong Village	15.82(25/158)^ab^	10.5–22.5
				Naqu Ranch	6.09(7/115)^de^	2.5–12.1
	Nyainrong	9.76(86/881)^b^	7.9–11.9	Gaque Ranch	10.62(24/226)^bcd^	1.8–4.0
				Payu Village	7.17(22/307)^de^	4.6–10.7
				Yumaoxiong Village	11.49(40/348)^bcd^	8.3–15.3
	Subtotal	9.86(166/1683)	8.5–11.4		9.86(166/1683)	8.5–11.4
Total		9.38(261/2784)	8.3–10.5		9.38(261/2784)	8.3–10.5

Note: The same lowercase letters within columns represent no significant differences between groups (*p* > 0.05) and different lowercase letters within columns represent significant differences between groups (*p* < 0.05).

### Seroprevalence of *T. gondii* in yaks by year

The seroprevalence of *T. gondii* in yaks of the Qinghai–Tibet Plateau in 2018 and 2019 was 8.35% (103/1233; 95% CI, 6.9–10.0) and 10.19% (158/1551; 95% CI, 8.7–11.8), respectively, displaying no significant difference between years (*p* > 0.05).

The prevalence of *T. gondii* in yaks from Qinghai Province in 2018 and 2019 was 8.60% (41/477; 95% CI, 6.2–11.5) and 8.65% (54/624; 95% CI, 5.5–14.1), respectively, with no significant difference (*p* > 0.05). However, in the Tibet Autonomous Region, the prevalence rates were 8.20% (62/756; 95% CI, 6.4–10.4) and 11.22% (104/927; 95% CI, 9.3–13.4), in 2018 and 2019, respectively which shows a significant difference between years (*p* < 0.05).

Positive rates at different sampling sites in Qinghai ranged from 0% (0/11) to 19.79% (19/96) in 2018 and from 7.54% (15/199) to 12.20% (5/41) in 2019, whereas, in Tibet, positivity rates ranged from 6.09% (7/115) to 12.34% (19/154) in 2018 and from 5.00% (5/100) to 15.82% (25/158) in 2019. Four sampling sites were sampled in 2018 and 2019, showing positive rates of 3.66% and 8.56% in Laozhaxi Ranch, 11.72% and 9.18% in Gaque Ranch, 9.04% and 14.38% in Yumaoxiong village, but 2.34% and 13.24% in Payu village, showing highly significant differences (*p* < 0.01) (Table 2).

**Table 2:** Seroprevalence of *T. gondii* infection by year in yaks on the Qinghai–Tibetan Plateau.

Provinces	Counties	Sampling sites	2018		2019		Average	
			Positive rates% (No. positive/No. samples)	95% CI	Positive rates% (No. positive/No. samples)	95% CI	Positive rates% (No. positive/No. samples)	95% CI
Qinghai	Datong	Datong Ranch	19.79(19/96)^a^	12.4–29.2	–	–	19.79(19/96)^a^	12.4–29.2
	Gangcha	Qingqingcao Ranch	5.41(4/74)^cde^	1.5–13.3	–	–	5.41(4/74)^bd^	1.5–13.3
	Guinan	Laozhaxi Ranch	3.66(3/82)^de^	0.8–10.3	8.56(16/187)^bcd^	4.1–26.2	7.06(19/269)^cd^	4.3–10.8
	Huangzhong	Slaughterhouses	17.02(8/47)^ab^	7.7–30.8	–	–	14.77(13/88)^ab^	8.1–23.9
		Fengtai Ranch	–	–	12.20(5/41)^abcd^	4.3–12.1		
	Qilian	Qilain Ranch	4.19(7/167)^de^	1.7–8.5	–	–	6.01(22/366)^d^	3.8–9.0
		Warinai Ranch	–	–	7.54(15/199)^cd^	9.3–13.4		
	Wulan	Chaidamu Ranch	0.00(0/11)^abcde^	0.0–28.5	–	–	8.65(18/208)^bcd^	5.2–13.3
		Tongpu Ranch	–	–	9.14(18/197)^abcd^	5.0–13.5		
	Total		8.60(41/477)	6.2–11.5	8.65(54/624)	5.5–14.1	8.63(95/1101)	7.0–10.5
Tibet	Seni	Dasa Village	–	–	8.07(13/161)^bcd^	4.4–13.4	9.98(80/802)^bc^	8.0–12.3
		Gaerde Ranch	–	–	9.65(11/114)^abcd^	4.9–16.6		
		Maqing Village	12.34(19/154)^abc^	7.6–18.6	–	–		
		Mufa Ranch	–	–	5.00(5/100)^d^	1.6–11.3		
		Namaqiegong Village	–	–	15.82(25/158)^a^	10.5–22.5		
		Naqu Ranch	6.09(7/115)^cde^	2.5–12.1	–	–		
	Nyainrong	Gaque Ranch	11.72(15/128)^abc^	6.7–18.6	9.18(9/98)^abcd^	4.3–16.7	9.76(86/881)^bc^	7.9–11.9
		Payu Village	2.34(4/171)^e^	0.6–5.9	13.24(18/136)^abc^	8.0–20.1		
		Yumaoxiong Village	9.04(17/188)^bcd^	5.4–14.1	14.38(23/160)^ab^	9.3–20.8		
	Total		8.20(62/756)	6.4–10.4	11.22(104/927)	9.3–13.4	9.86(166/1683)	8.5–11.4
Qinghai and Tibet			8.35(103/1233)	6.9–10.0	10.19(158/1551)	8.7–11.8	9.38(261/2784)	8.3–10.5

Note: The same lowercase letters within columns represent no significant differences between groups (*p* > 0.05) and different lowercase letters within columns represent significant differences between groups (*p* < 0.05).

### Seroprevalence of *T. gondii* in yaks by sex

The data in Table 3 show that the positive rates of *T. gondii* in male and female yaks of the Qinghai–Tibetan Plateau were 8.71% (89/1 022; 95% CI, 4.6–7.0) and 9.76% (172/1762; 95% CI, 8.4–11.2), respectively, which showed no significant difference between the sexes (*p* > 0.05). The positive rates of *T. gondii* in male and female yaks in Qinghai Province were 7.13% (35/505; 95% CI, 4.9–9.5) and 9.90% (59/596; 95% CI, 7.6–12.6), respectively. In the Tibet Autonomous Region, the percentages were 10.25% (53/517; 95% CI, 7.8–13.2) and 9.69% (113/1166; 95% CI, 8.1–11.5), respectively, with no significant difference between the sexes (*p* > 0.05) (Table 3).

**Table 3:** Seroprevalence of *T. gondii* infection by sex in yaks on the Qinghai–Tibetan Plateau in 2018 and 2019.

	Qinghai						Tibet						Qinghai and Tibet					
Sex	2018		2019		Total		2018		2019		Total		2018		2019		Total	
	Positive rates % (No. positive/No. samples)	95% CI	Positive rates % (No. positive/No. samples)	95% CI	Positive rates % (No. positive/No. samples)	95% CI	Positive rates % (No. positive/No. samples)	95% CI	Positive rates % (No. positive/No. samples)	95% CI	Positive rates % (No. positive/No. samples)	95% CI	Positive rates % (No. positive /No. sample)	95% CI	Positive rates % (No. positive /No. sample)	95% CI	Positive rates % (No. positive /No. sample)	95% CI
Male	6.15(15/244)^a^	3.5–9.9	8.05(21/261)^a^	5.1–12	7.13(35/505)^a^	4.9–9.5	8.57(12/140)^a^	4.5–14.5	10.88(41/377)^a^	7.9–14.5	10.25(53/517)^a^	7.8–13.2	7.03(27/384)^a^	4.7–10.1	9.72(62/638)^a^	7.5–12.3	8.71(89/1022)^a^	4.6–7.0
Female	11.16(26/233)^a^	7.4–15.9	9.09(33/363)^a^	6.3–12.5	9.90(59/596)^a^	7.6–12.6	8.12(50/616)^a^	6.1–10.6	11.45(63/550)^a^	7.9–14.5	9.69(113/1166)^a^	8.1–11.5	8.95(76/849)^a^	7.1–11.1	10.51(96/913)^a^	8.6–12.7	9.76(172/1762)^a^	8.4–11.2
Total	8.60(41/477)	6.2–11.5	8.65(54/624)	6.6–11.1	8.63(95/1101)	7.0–10.5	8.20(62/756)	6.4–10.4	11.22(104/927)	9.3–13.4	9.86(166/1683)	8.5–11.4	8.35(103/1233)	6.9–10.0	10.19(158/1551)	8.7–11.8	9.38(261/2784)	8.3–10.5

Note: The same lowercase letters within columns represent no significant differences between groups (*p* > 0.05) and different lowercase letters within columns represent significant differences between groups (*p* < 0.05).

### Seroprevalence of *T. gondii* in yaks by age

In this study, the yaks were divided into five age groups. The highest positive rate of *T. gondii* in yaks was found at the age of >7 years old (11.76%, 28/238; 95% CI, 8.0–16.6). However, a relatively low prevalence rate was found at 0 < year ≤ 1 (5.59%, 16/286; 95% CI, 3.2–8.9), which showed a significant difference (*p* < 0.05) at different ages. In Qinghai Province, the highest positive rate was found at the age of 5 < year ≤ 7 (17.88%, 27/151; 95% CI, 12.1–24.9), and the lowest positive rate was found at 0 < year ≤ 1 (4.71%, 12/255; 95% CI, 2.5–8.1), showing a significant difference (*p* < 0.05). The highest positive rate of *T. gondii* in yaks in the Tibet Autonomous Region was found at the age of 0 < year ≤ 1 (12.90%, 4/31; 95% CI, 3.6–29.8), and the lowest rate was observed at 3 < year ≤ 5 (8.88%, 49/552; 95% CI, 6.6–11.6). There was no significant difference found among different ages of yaks in the region (*p* > 0.05) (Table 4).

**Table 4:** Seroprevalence of *T. gondii* infection by age in yaks on the Qinghai–Tibetan Plateau in 2018 and 2019.

Ages	Qinghai						Tibet						Qinghai and Tibet					
	2018		2019		Subtotal		2018		2019		Subtotal		2018		2019		Subtotal	
	Positive rates % (No. positive/No. samples)	95% CI	Positive rates % (No. positive/No. samples)	95% CI	Positive rates % (No. positive/No. samples)	95% CI	Positive rates % (No. positive/No. samples)	95% CI	Positive rates % (No. positive/No. samples)	95% CI	Positive rates % (No. positive/No. samples)	95% CI	Positive rates % (No. positive /No. sample)	95% CI	Positive rates % (No. positive /No. sample)	95% CI	Positive rates % (No. positive /No. sample)	95% CI
0＜year≤1	6.61(8/121)^bc^	2.9–12.6	2.99(4/134)^c^	0.8–7.5	4.71(12/255)^b^	2.5–8.1	5.00(1/20)^a^	0.1–24.9	27.27(3/11)^a^	6.0–61.0	12.90(4/31)^a^	3.6–29.8	6.38(9/141)^a^	3.0–11.8	4.83(7/145)^c^	2.0–9.7	5.59(16/286)^b^	3.2–8.9
1＜year≤3	7.76(9/116)^bc^	3.6–14.2	7.76(17/219)^bc^	4.6–12.1	7.76(26/335)^b^	5.1–11.2	9.59(14/146)^a^	5.3–15.6	11.76(26/221)^a^	7.8–16.8	10.90(40/367)^a^	7.9–14.5	8.78(23/262)^a^	5.7–12.9	9.77(43/440)^abc^	7.2–12.9	9.40(66/702)^a^	7.4–11.8
3＜year≤5	3.42(4/117)^c^	0.9–8.5	8.61(13/151)^b^	4.7–14.3	6.34(17/268)^b^	3.7–10.0	7.98(19/238)^a^	4.9–12.2	9.55(30/314)^a^	6.54–13.4	8.88(49/552)^a^	6.6–11.6	6.48(23/355)^a^	4.2–9.6	9.25(43/465)^bc^	6.8–12.3	8.05(66/820)^ab^	6.3–10.1
5＜year≤7	17.78(16/90)^a^	10.5–27.3	18.03(11/61)^a^	9.4–30.0	17.88(27/151)^a^	12.1–24.9	6.81(19/279)^a^	4.2–10.4	12.66(39/308)^a^	9.2–16.9	9.88(58/587)^a^	7.6–12.6	9.49(35/369)^a^	6.7–12.9	13.55(50/369)^a^	10.2–17.5	11.52(85/738)^a^	9.3–14.0
year＞7	12.12(4/33)^ab^	3.4–28.2	15.25(9/59)^ab^	7.2–27.0	14.13(13/92)^a^	4.7–14.3	12.33(9/73)^a^	5.8–22.1	8.22(6/73)^a^	3.1–17.0	10.27(15/146)^a^	5.9–16.4	12.26(13/106)^a^	6.7–20.0	11.36(15/132)^ab^	6.5–18.1	11.76(28/238)^a^	8.0–16.6
Total	8.60(41/477)	6.2–11.5	8.65(54/624)	6.6–11.1	8.63(95/1101)	7.0–10.5	8.20(62/756)	6.4–10.4	11.22(103/927)	9.3–13.4	9.86(166/1683)	8.5–11.4	8.35(103/1233)	6.9–10.0	10.19(158/1551)	8.7–11.8	9.38(261/2784)	8.3–10.5

Note: The same lowercase letters within columns represent no significant differences between groups (*p* > 0.05) and different lowercase letters within columns represent significant differences between groups (*p* < 0.05).

## Discussion

To date, there are insufficient data on yak toxoplasmosis, and there are only a limited number of reports on yak toxoplasmosis from China. As a globally important zoonotic parasite, *T. gondii* was first isolated from humans in the 1930s and is estimated to have infected 30% of the world’s population [24]. The transmission of the parasite to humans can occur through ingestion of raw or inadequately cooked infected meat from domestic animals or through consumption of animal products containing *T. gondii* cysts. In China, especially on the Qinghai–Tibetan Plateau, *T. gondii* infection in yaks is an important risk factor for the local population. Therefore, studying the prevalence of *T. gondii* in yaks is an important step to control foodborne toxoplasmosis in these regions.

Evaluation by serological tests, especially ELISA, is a convenient and efficient method to detect *T. gondii* infection in animals [9]. In the present study, the overall prevalence of *T. gondii* was found to be 9.38%, which is slightly lower than in the previous study [15]. Dubey reported in 2008 that the estimated overall global prevalence of *T. gondii* in cattle using different detection methods was 14.96% (8 286/55 377) [7]. According to survey results from 2000 to 2017, the overall prevalence of *T. gondii* in cattle in China was 10.6% (2 781/26 210), with total prevalence in yaks of 13.5% (1221/9042) [5]. Compared to other countries, the prevalence of *T. gondii* in yaks on the plateau was lower than in cattle in Brazil (54.4%, 272/500) and Estonia (18.6%, 743/3991) [3, 12]; however, slightly higher than 7.3% (31/422) in cattle in Japan and 3.2% (127/4033) in Poland [11, 19]. The reason for this difference may be related to differences in detection methods, climate, environment and geographical factors, as well as effective pest control measures and level of breeding management.

The positive prevalence rate of *T. gondii* in yaks in Qinghai Province and the Tibet Autonomous Region showed no significant difference (*p* > 0.05). Among the eight counties, Datong County had the highest positive rate compared to Gangcha County, which showed no significant difference (*p* < 0.05). At the 18 sampling sites, all showed toxoplasma antibodies, except one. In these counties, the prevalence rate ranged from 0.0% to 19.4%, showing a significant difference (*p* < 0.05) between sampling sites. The difference in positive prevalence rates may be due to pasture management, climate, and environmental conditions associated with these areas as risk factors. The altitude of the different sampling sites also affects the infection rate of the parasite: for example, the positive rate of *T. gondii* infection was higher in yaks at Maqing Village (4480 m above sea level) and Namaqiegong Village (4530 m above sea level) than that at Payu Village (4970 m above sea level). Some studies have shown that *Toxoplasma* infections are often high in areas of the world with hot, humid climates and lower altitudes because the oocysts survive better in these environments [1, 6].

In the present study between 2018 and 2019, the seroprevalence of *T. gondii* on both the plateau and in Qinghai Province showed no significant difference (*p* > 0.05) between years. However, in Tibet, the prevalence rate showed a significant difference between 2018 and 2019 (*p* < 0.05). A previous study conducted by Li *et al.* [15] in 2012 and 2013 reported seroprevalence of 20.5% (142/693) and 26.7% (129/484) in yaks on the plateau. The discrepancies between the present and previous studies may be due to *T. gondii* detection methods or sampling locations. In addition, we found that of the four sampling sites in these two years, three sites had higher positive rates in 2019 compared to 2018, although the difference was not significant (*p* > 0.05). A possible reason for this could be the collection of samples in two different seasons, as in 2018 the samples were collected in winter, while in 2019 the samples were collected in summer. Yaks can become infected through contact with contaminated grass, water or soil contaminated with *T. gondii* oocysts. In our study, the positive rates of samples collected in summer were higher than those collected in winter, suggesting that fresh grass used as yak feed may contain *T. gondii* oocysts and the moist environment is favourable for oocyst survival. In a previous study, it was reported that *T. gondii* infection tends to be in warm and humid environments due to different climatic and geographical conditions, which was consistent with our findings [13].

In terms of sex, we found that the *T. gondii* positive rate was higher in female yaks than in males, but the difference was not significant (*p* > 0.05). Previous studies have concluded that sex is not necessarily a risk factor associated with *T. gondii* infection [25]. These findings are consistent with our current results, indicating that *T. gondii* infection does not have a particular sex specificity.

We found that age was an important factor influencing *T. gondii* infection in yaks. In this study, significant differences in the prevalence of *T. gondii* were observed between different age groups of yaks (*p* < 0.05). Our results showed that, as the age of yaks increases, the prevalence of *T. gondii* gradually increased. As adult yaks graze freely, they are more likely to be exposed to food and water contaminated with *T. gondii* oocysts. Previous studies also reported that the seroprevalence of *T. gondii* increased with the age of the yaks [18].

In Qinghai and Tibet, yaks are the main source of meat and milk for the local population. However, when raw yak meat and their internal organs, in which *T. gondii* cysts are present, are eaten by local cats and other Felidae animals, they can become infected and *T. gondii* oocysts are produced and shed. The Yangtze River and Yellow River in China originate from the Qinghai–Tibetan Plateau. *Toxoplasma gondii* oocysts can survive in water and can be transported to downstream areas and even the ocean via freshwater rivers, posing a great threat to humans and animals living along riverbanks [8]. If *T. gondii* infection from these sources is not effectively controlled, it will become a public health risk factor in downstream areas and affect the prevention and control of *T. gondii* infection in these areas. Therefore, our study provides basic data for local and downstream public health prevention of zoonotic toxoplasmosis.

## Conclusions

The present study concluded that climate, geographical conditions, and age were the major risk factors associated with *T. gondii* infection in yaks. The results indicate that yaks infected with *T. gondii* are likely to pose a potential threat to humans in the region. This study provides data for the prevention and control of *T. gondii* infection on yak farms on the Qinghai–Tibetan Plateau and the public health threat to local residents and generally in China.

## Competing interests

The authors declare that they have no competing interests.

## Funding information

This study was supported in part by the National Key Research and Development Program of China (Grant No. 2018YFD0502305), National Risk Assessment Project for Quality and Safety of Agricultural Products (Grant No. GJFP2019027), Shanghai Science and Technology Commission Scientific Research Project (Grant No. 20140900400), and Shanghai Agriculture Applied Technology Development Program, China (Grant No. 2019-02-08-00-08-F01151).
